# When to shed? Patterns and drivers of time to first ecdysis in snakes

**DOI:** 10.1002/ece3.10364

**Published:** 2023-08-02

**Authors:** Cecilia Wagner, Ashadee K. Miller, Hanlie M. Engelbrecht, Harry W. Greene, Graham J. Alexander

**Affiliations:** ^1^ School of Animal, Plant and Environmental Sciences University of the Witwatersrand Johannesburg South Africa; ^2^ Department of Ecology and Evolutionary Biology, Corson Hall Cornell University Ithaca New York USA

**Keywords:** ancestral reconstruction, biosensors, chemical crypsis, life history, maternal care, slough

## Abstract

Time from birth or hatching to the first shed (postnatal ecdysis) in snakes ranges from about an hour to several weeks depending upon the species. We assessed patterns in time to postnatal ecdysis in 102 snake species for which we could source appropriate information, covering 2.6% of all extant snake species, and related measures to various biological traits. Reconstruction revealed ancestral time to postnatal ecdysis to be 11 days. Since time to postnatal ecdysis can be shorter or longer than the ancestral state, we argue that there are several competing drivers for time to postnatal ecdysis. A reduced time to postnatal ecdysis has evolved in several lineages, commonly in ambush‐foraging, viviparous vipers, while extended time to postnatal ecdysis is associated with oviparous species with maternal care. Of central importance is the impact of postnatal ecdysis on the scent levels of neonates, resulting in a reduction of time to postnatal ecdysis in chemically cryptic species, while the pivotal role of scent in mother–neonate recognition has resulted in the retention or extension of time to postnatal ecdysis. We showed that postnatal ecdysis improves chemical crypsis. The patterns revealed in this study suggest that measures of time to postnatal ecdysis can provide insights into the biology of snakes and be used as an indicator of certain life history traits.

## Graphical abstract

We assessed patterns in time to postnatal ecdysis in snake species, and related measures to various biological traits. Time to postnatal ecdysis can be shorter or longer than the ancestral state, which we estimated at 8–9 days, indicating that there are several competing drivers for time to postnatal ecdysis. We showed that postnatal ecdysis improves chemical crypsis and that measures of time to postnatal ecdysis can provide valuable insights into snake biology.

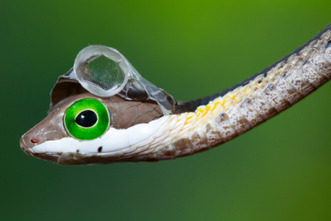

## 1 INTRODUCTION

Ecdysis, the shedding of the epidermis in a discrete event to accommodate skin renewal and growth (Tu et al., 2002), is a characteristic typical of snakes (Lillywhite & Coleman, 2016; Lillywhite & Maderson, 1982). Frequency of shedding is affected by temperature, humidity, food intake and reproductive behaviour (Alexander, 2018; Carlson et al., 2014; Cliburn, 1976; Gibson et al., 1989; Greene et al., 2002; Semlitch, 1979) but appears to also be under some measure of endogenous control (Alexander & Brooks, 1999). Shedding can affect foraging and thermoregulatory behaviours. For instance, during the ‘blue’ preshedding phase, snakes generally do not forage and are more reclusive, which may result in lower body temperatures (*T*_b_) due to a reluctance to bask (Kitchell, 1969). However, in some situations, snakes may select higher *T*_b_s during the blue phase to promote new epidermal growth (Brown et al., 1982; Gibson et al., 1989). Vulnerability to predation during this phase also increases because of the snake's reduced visual acuity, and during the actual shedding event, due to compromised chemical and visual crypsis (Miller et al., 2015). The loss of the shed skin also represents a significant loss of energy, with measures ranging from 3% to 11% of the snake's annualised metabolic expenditure, depending on lifestyle and body size (Alexander, 1996; Smith, 1976). The shedding cycle in snakes is thus important to many aspects of their life history and ecology.

In snakes, the first shedding event, known as postnatal ecdysis (PNE), occurs anywhere between an hour and several weeks after birth or hatching (Ball, 2004; Greene, 1997). Neonatal skin is more permeable to water than adult skin and thus neonates tend to seek humid microenvironments and are more reclusive before PNE (Agugliaro & Reinert, 2005; Graves et al., 1986; Tu et al., 2002). After PNE, lipid content in neonatal skin increases, which results in the skin becoming less permeable, reducing cutaneous water loss (Agugliaro & Reinert, 2005; Ball, 2004; Graves et al., 1986; Tu et al., 2002). Postnatal ecdysis also plays a role in scent recognition. For example, *Coronella austriaca* (smooth snakes) have increased chemosensory responses to nonkin conspecifics and prey scents after PNE (Pernetta et al., 2009). Only after PNE do neonates typically initiate feeding and, in species with maternal care of young, disperse from the birthing place (Alexander, 2018; Dwyer et al., 2019; Greene et al., 2002). The wide interspecific variation in time to PNE and its impact on water balance, feeding and growth in neonates suggests that it is likely to be under strong natural selection.

Neonate *Bitis arietans* (puff adders) shed within hours of birth (GJA, AKM, personal observation). These ambush‐foraging vipers employ chemical crypsis to avoid olfactory detection by macrosmatic predators (Miller, 2022; Miller et al., 2015). Although puff adder shed skin is odorous and easily detectable by predators, puff adders maintain their crypsis by moving to a new location immediately after shedding (Miller et al., 2015). Similarly, fluids produced at parturition are likely to be highly odorous (Butler et al., 1995), and both puff adder mothers and their offspring may consequently experience increased risk of predation at this time. This parturition‐associated compromise in chemical crypsis may explain the short time to PNE in this species since PNE could reduce detectability by macrosmatic predators. To date, puff adders are the only terrestrial vertebrates known to be chemically cryptic, but it is likely that other ambush‐foraging viperids that are preyed upon by macrosmatic predators also possess some form of chemical crypsis (Miller et al., 2015). It is thus possible that the short time to PNE in puff adders has evolved in concert with chemical crypsis as an antipredator adaptation.

We collected measures of time to PNE for a diverse range of snake species and evaluated how this related to their phylogeny, ecology, biogeography and life history traits to evaluate what could be driving changes in time to PNE. We assessed this variation at various nodes in the phylogeny and estimated the ancestral PNE using ancestral state reconstruction analysis. We also assessed which of several morphological, ecological, reproductive, behavioural and biogeographic traits best explained the observed patterns in time to PNE for our dataset using analysis of variance. Lastly, we directly tested the hypothesis that PNE reduced the scent levels of neonate puff adders, using trained scent‐matching dogs as biosensors. There are several candidate selective forces that may impact time to PNE, some acting to reduce time to PNE and others acting to extend it. This theoretical framework generated several hypotheses and sets the scene for several future investigations.

## 2 MATERIALS AND METHODS

### 2.1 Postnatal ecdysis data collection and categorisation

Records of time to PNE were collected for snakes through data mining of the scientific literature and consultation with herpetologists and snake breeders. The literature reporting PNE records were sourced by searching Google Scholar using the following keywords: ‘ecdysis’, ‘postnatal’, ‘snake’, ‘neonate’, ‘shed’, ‘moult’, ‘slough’, ‘skin’, ‘hatch’, ‘birth’, ‘captive’, ‘reproduction’, ‘breed’ and ‘husbandry’. Records were mostly reported as a range of days to PNE observed within and across clutches or litters from one or more sources. The average number of days to PNE was calculated by averaging the time to PNE for each record for each species.

**FIGURE 1:**
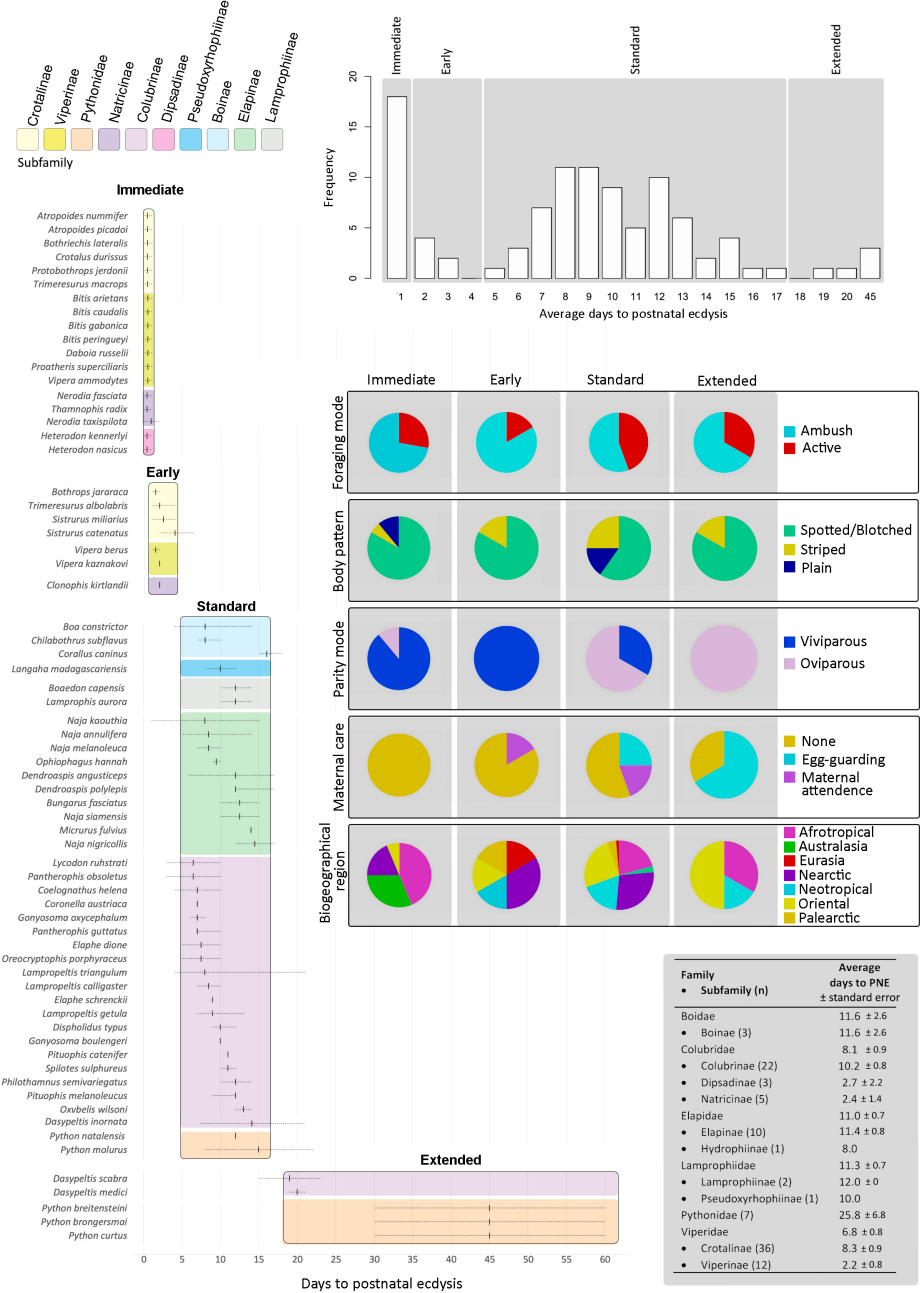
Trimodal distribution of average days to postnatal ecdysis for sampled species, dividing data into immediate PNE (1 ≤ day), early PNE (2–4 days), standard PNE (5–17 days) and extended PNE (>17 days). Pie charts represent how life history traits are apportioned among species (*n* = 102) within each PNE category. Range and average of time to PNE shown per sampled species and grouped by subfamily. Table insert: average number of days to postnatal ecdysis (PNE), with standard errors, for species included in this study grouped by family and subfamily. Numbers in brackets in the left column refer to the number of species in the data set.

Records of time to PNE for 102 Afrophidia species (92 Caenophidia and 10 Henophidia species, Harrington & Reeder, 2017) were gathered, of which 17 were collected through personal communication or unpublished data (B. Newman, S. Forum, A. K. Miller, A. Roberts, ReptileForum; G. J. Alexander, B. Vermeulen, C. Rowberry, Toronto Zoo, J. M. Barends, B. Baldwin, I. Recchio, Alexander Herp Lab, San Diego Zoo, C. Mezsar, J. K. Warner, & M. Vermeulen) and 85 based on the available literature (Table S1). A frequency histogram showing the distribution of records of time to PNE was trimodal (Figure 1). However, a high proportion of species were reported to shed almost immediately after birth, and this subset was considered to be a different category to those that shed during subsequent days. Thus, the species were assigned to four categories: immediate PNE (≤1 day); early PNE (2–4 days); standard PNE (5–17 days); and extended PNE (>17 days).

### 2.2 Ancestral state reconstruction

The ancestral time to PNE was estimated for the species in the data set using a likelihood‐based ancestral state reconstruction (ASR) analysis to assess how time to PNE may have evolved. We based our ASR analysis on the phylogenetic tree produced by Tonini et al. (2016), of which we trimmed the tree tips to accommodate for our data set of 102 Afrophidia species. The analysis was made using ‘phytools’ v. 1.5–1, with the ‘anc.ML’ function and the Ornstein–Uhlenbeck (OU) model, and the average days to PNE as a continuous character (Revell, 2012). The OU model was chosen as it had a better AIC value (686.0) than the BM model (704.8), and assumes that the trait values are influenced not only by random factors, but also by deterministic factors. Therefore, the OU model allows for our postulation that there are several competing drivers for time to postnatal ecdysis. To estimate the number of character state changes between our theoretical PNE categories (immediate PNE, early PNE, standard PN and extended PNE), stochastic character mapping was calculated with the ‘make.simmap’ function and all‐rates‐different (ARD) model.

### 2.3 Analysis of life history traits

Information related to several life history traits were collected from the literature and online resources (Table S2). These included (categories defined below): foraging mode, body pattern, parity mode, maternal care, biogeographic region and subfamily. Foraging mode was categorised as either ambush or active foraging. The level of camouflage in body pattern was loosely based on Allen et al. (2013) and consisted of ‘spotted‐blotched’ (separate spots or blended blotches; cryptic while motionless), ‘striped’ (longitudinal or transverse striped; cryptic during rapid locomotion) and ‘plain’ (uniform colour from a distance). Parity mode was classified as either ‘oviparous’ or ‘viviparous’. Maternal care was categorised as ‘none’, ‘egg‐guarding’ or ‘maternal attendance’ of young. Biogeographic regions were categorised based on Wallace (1876).

A stepwise, factorial analysis of variance (ANOVA) was used to assess whether the four categories of PNE (dependent variable) were related to life history traits (independent variables). Average days to PNE were categorised and assigned numerically, thereby transforming it to ordinal categorical data. Assumptions of normal distribution, homogeneity of variance and independence of observations were verified. Multicollinearity between variables was tested through the variance inflation factor (VIF), and all interactions between independent variables were initially included. Due to the collinearity between foraging mode and body pattern (in the data set and in theory), we combined these variables into a single composite category for the analysis (foraging mode/pattern). Stepwise Akaike Information Criteria (AIC) was measured to produce the optimal set of traits. A post hoc Tukey's test was calculated to indicate which traits were significant at α < .05. The above statistical analyses were performed in R version 3.0.3 using RStudio version 1.2.1335 (R Core Team, 2019; RStudio Team, 2019), using ‘car' (Fox & Weisberg, 2019) and ‘MASS’ (Venables & Ripley, 2002) packages.

To accommodate the nonindependence of our species and their life history data, a phylogenetically informed ANOVA was performed, using the ‘phylANOVA’ function in ‘phytools’.

### 2.4 The effect of PNE on neonate chemical crypsis

Scent‐matching dogs (*n* = 6) were trained and scent trials performed in accordance with established methods published previously (Miller et al., 2015). Briefly, dogs were provided with a reference scent and tasked to find its twin within a line‐up of blanks (unscented cotton cloths) and environmental controls (cotton cloths scented with appropriate background odours; see Miller et al., 2015). The chemical detectability of neonate puff adders (*n* = 20) was tested using dogs at three specific time frames postbirth (pre‐PNE, immediately post‐PNE, 2 weeks post‐PNE). Data were arcsine transformed and analysed using a one‐factor ANOVA and compared with a random‐choice model, based on a 1:6 target:nontarget cloth frequency. Significant differences between treatments were assessed using post hoc Tukey's tests. Analyses were performed using STATISTICA v.8 (STATISTICA Data Analysis Software System 2001, http://www.statsoft.com).

## 3 RESULTS

### 3.1 Postnatal ecdysis data overview

**FIGURE 2:**
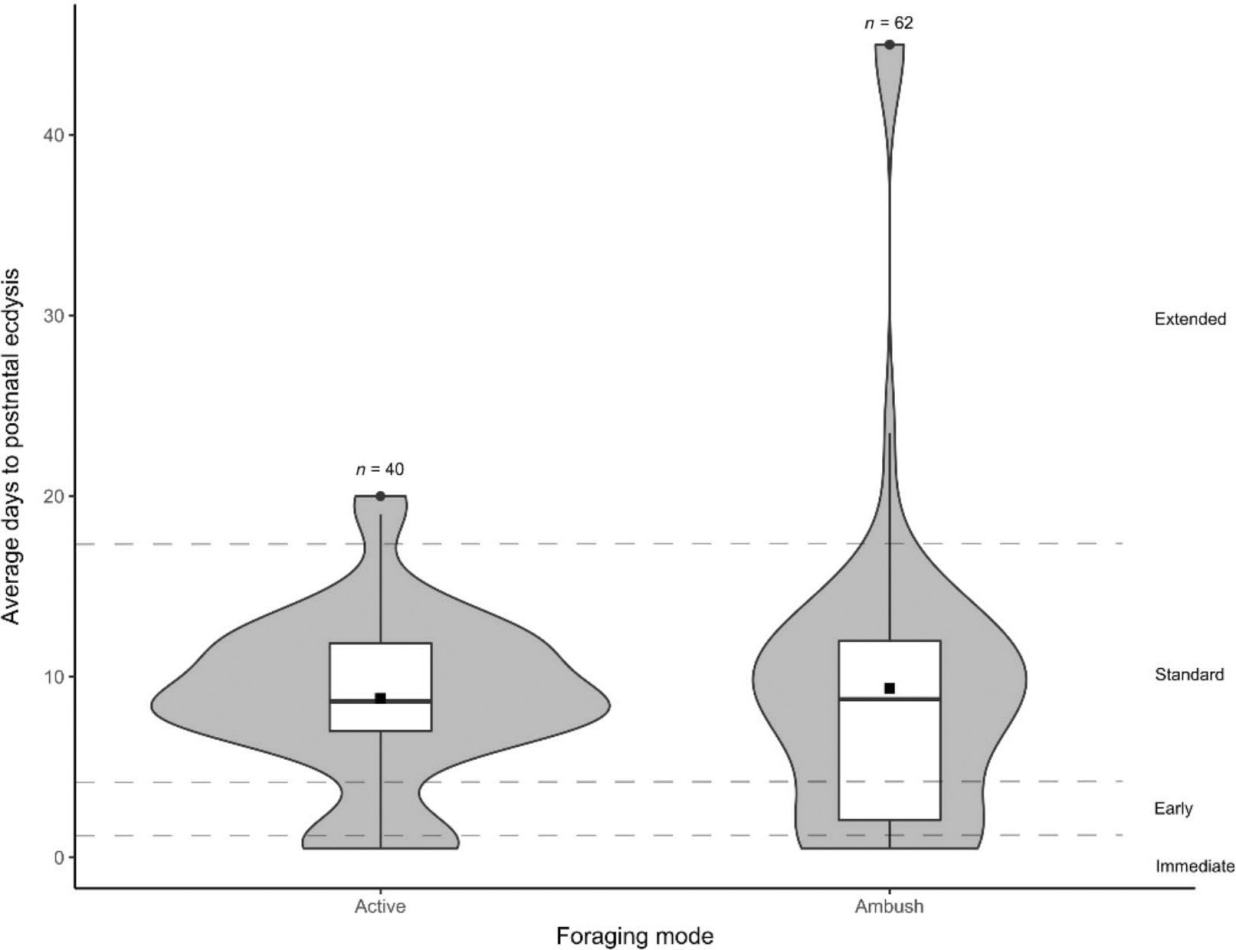
Violin boxplots of the average days to postnatal ecdysis (PNE) of sampled species with different foraging modes, namely active and ambush foraging. Immediate PNE (<1 day), early PNE (2–4 days), standard PNE (5–17 days) and extended PNE (>17 days) are separated by a dashed line. ▪—overall average days to PNE. *n*, total sampled species.

**FIGURE 3:**
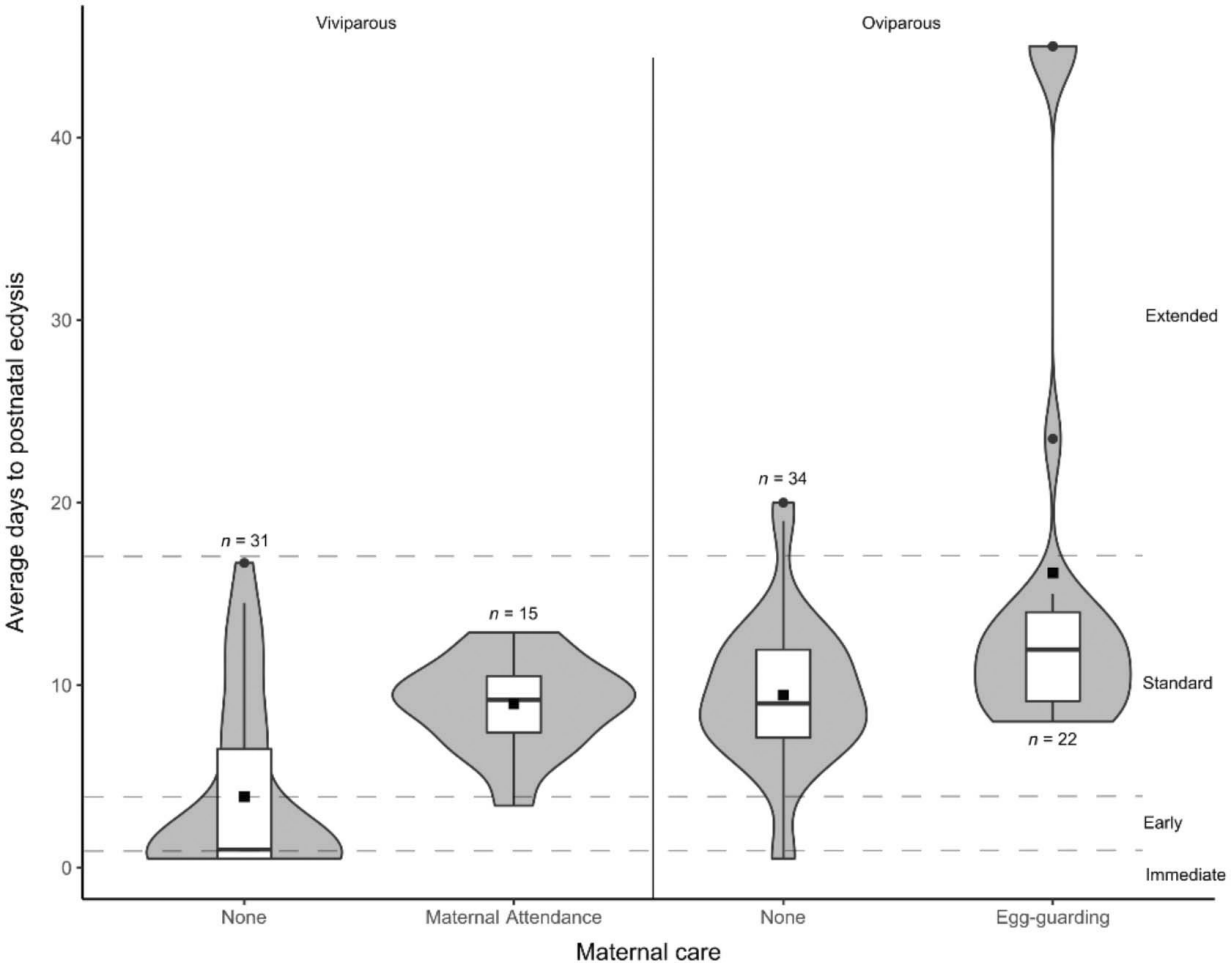
Violin boxplots of the average days to postnatal ecdysis (PNE) of sampled viviparous species that have maternal care or no parental care and sampled oviparous species that have egg‐guarding or no parental care. Immediate PNE (≤1 day), early PNE (2–4 days), standard PNE (5–17 days) and extended PNE (>17 days) are separated by a dashed line. ▪—overall average days to PNE. *n*, total sampled species.

Standard PNE was most common in our sample (71% of species), followed by immediate PNE (17%), early PNE (6%) and extended PNE (6%). Sampled species with immediate PNE (*n* = 18), were mostly ambush foragers (72%), with spotted‐blotched body patterns (83%), and viviparous parity (89%) with no parental care (100%), occurring in mostly the Nearctic (38%) and Afrotropical (21%) regions (Figures 1, 2, 3). Some subfamilies were more inclined to certain PNE durations. For instance, most Viperinae, Natricinae and Dipsadinae in our sample showed on average immediate PNE or early PNE, whereas extended PNE featured only in two Colubrinae species and three species of the family Pythonidae. All other subfamilies were predominantly standard PNE (Figure 1, table insert). Several obvious patterns were evident: for example, immediate and early PNE were particularly common in the Viperinae making up 58% and 17% of species, respectively.

### 3.2 Ancestral state reconstruction

**FIGURE 4:**
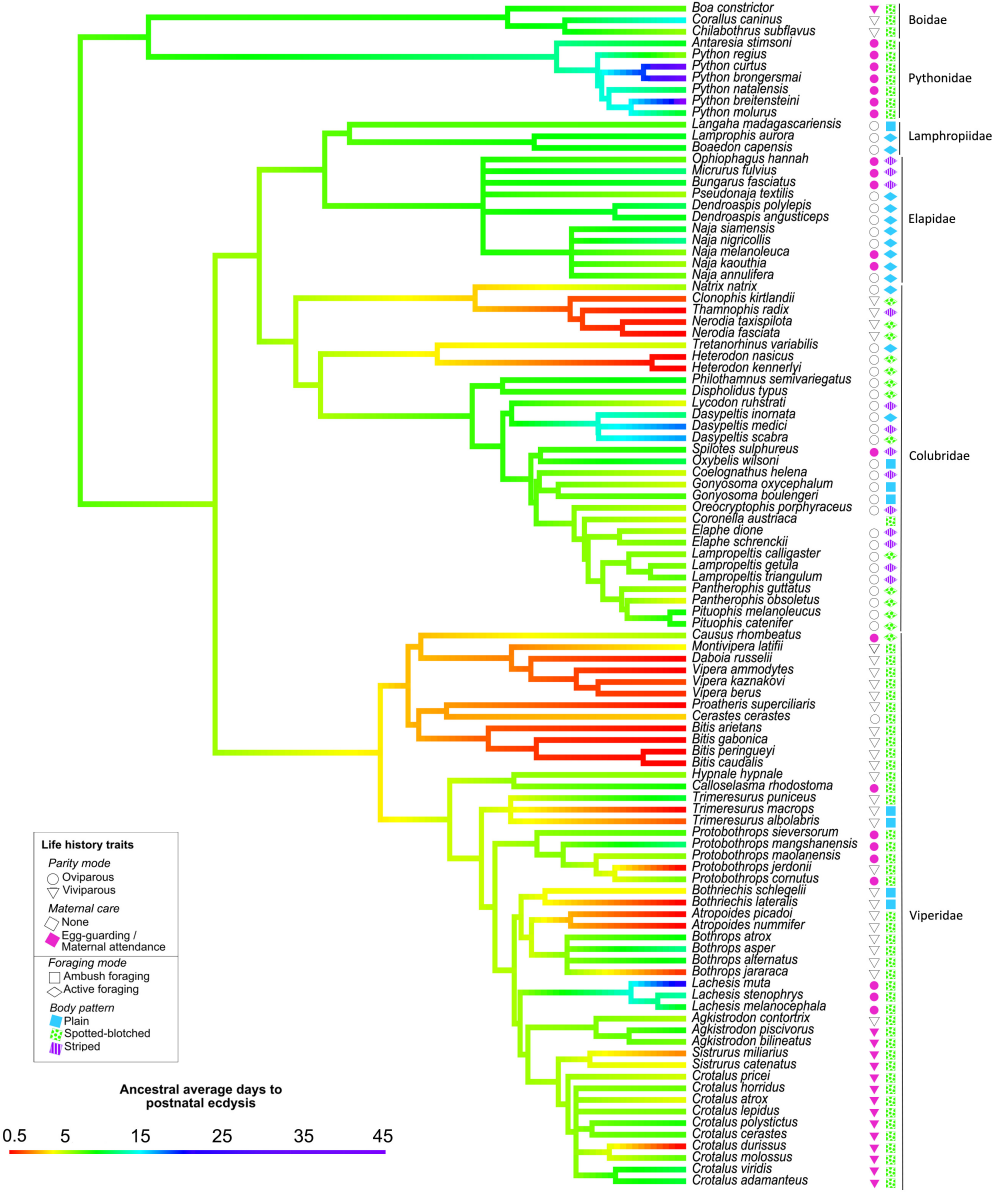
Ancestral state reconstruction tree based on 102 Afrophidae species (92 Caenophidia and 10 Henophidia species) describing the ancestral days to postnatal ecdysis illustrated on the branches by the colour gradient. Keys of life history traits (parity mode, maternal care, foraging mode and body pattern) are represented next to each species.

Ancestral state reconstruction revealed that the ancestral time to PNE was 11 days for our sampled Afrophidia species (CI: 2–20 days, based on BM model; Figure 4), and is thus taken to be standard PNE (5–17 days; Figure 1). Reductions in PNE (<5 days) were reconstructed for a total of 16 nodes and four clades, specifically within the Natricinae, Atropoides, Heterodon and Viperinae clades (Table A1 in Appendix 1). It is thus evident that a reduction in time to PNE has evolved multiple times independently within Caenophidia. Time to PNE has been extended (>17 days) in rare instances within Colubridae, Pythonidae and one known instance in Viperidae (*Lachesis muta*).

The mean number of changes between PNE categories (immediate PNE, early PNE, standard PNE, and extended PNE) were estimated through stochastic mapping (Table 1). It is evident that early PNE and extended PNE are fixed states, as early PNE never increases to standard or extended PNE and only decreases to immediate PNE, whereas extended PNE only ever decreases to the level of standard PNE, both with a mean number of 11 changes. Standard PNE seems viable to change in any direction, with a mean number of six to eight changes, which further supports the proposition that the ancestral state is standard PNE.

**TABLE 1:** Mean number of changes between postnatal ecdysis (PNE) categories (immediate PNE, early PNE, standard PNE and extended PNE) as estimated through stochastic mapping.

	Immediate PNE	Early PNE	Standard PNE	Extended PNE
Immediate PNE ‐>	0.0	6.1	4.5	0.0
Early PNE ‐>	10.7	0.0	0.0	0.0
Standard PNE ‐>	7.6	6.1	0.0	6.5
Extended PNE ‐>	0.0	0.0	11.2	0.0

### 3.3 Impact of biological traits on time to PNE

The analysis of variance (ANOVA) revealed several significant relationships between time to PNE and life history traits (*F*_39,62_ = 5.93; *p* < .001; adjusted *R* ^2^ = 0.66) for 102 Afrophidia species. Based on an AIC of −118.37, the set of traits that best explained variation in time to PNE were foraging mode/pattern (*F*_4,62_ = 3.88; *p* = .007), parity mode (*F*_1,62_ = 78.17; *p* < .001), maternal care (*F*_2,62_ = 21.21; *p* < .001), subfamily (*F*_10,62_ = 4.33; *p* < .001) and biogeographic region (*F*_6,62_ = 3.42; *p* = .005). Although parity mode was marginally multicollinear (VIF = 2.66), it was kept in the analysis as it improved model strength. Interactions between variables included foraging mode/pattern with biogeographic region, and maternal care with biogeographic region and subfamily. The post hoc Tukey's test revealed that time to PNE differed significantly between the following groups: plain ambushers and plain active foragers (*p* = .043); oviparous and viviparous modes (*p* < .001); maternal care and egg‐guarding, and no maternal care (*p* < .001); Neotropical and Nearctic regions (*p* < .030); Dipsadinae and Boinae, Colubrinae, Crotalinae, Elapinae, Natricinae, and Pythonidae (in order: *p* = .006, *p* < .001, *p* = .002, *p* = .005, *p* = .019, *p* < .001).

The phylANOVA indicated that time to PNE is significantly affected by parity mode (*F*_1,62_ = 34.83; *p* = .001), maternal care (*F*_2,62_ = 9.39; *p* = .02) and subfamily (*F*_10,62_ = 6.51; *p* = .01). The life history traits ‘biogeography’ (*F*_6,62_ = 0.61; *p* = .95) and ‘foraging mode/pattern’ (*F*_4,62_ = 1.36; *p* = .97) were not deemed significant.

### 3.4 The effect of PNE on neonate chemical crypsis

**FIGURE 5:**
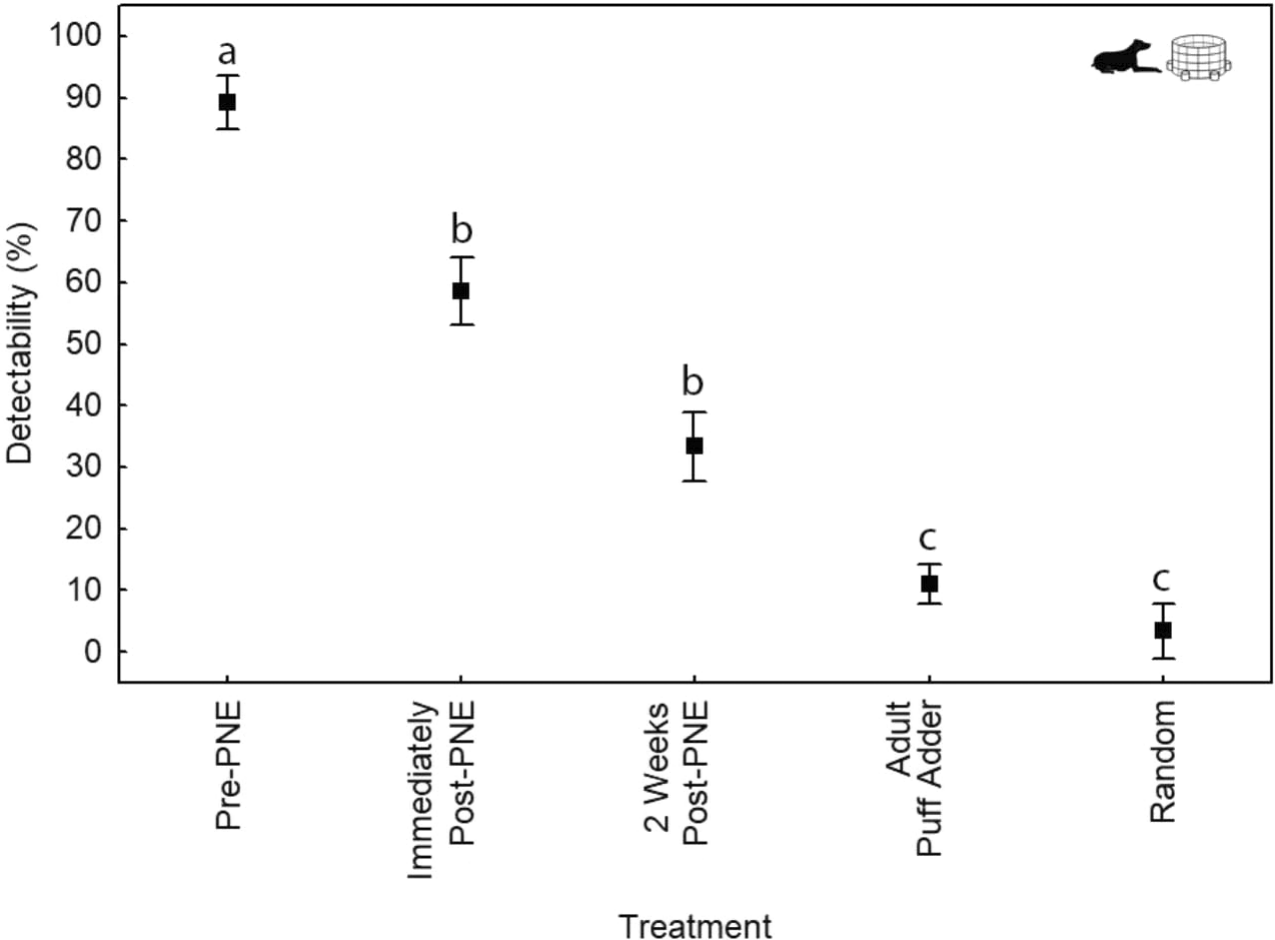
Success rates of dogs detecting puff adder scent (mean ± SE) for neonate puff adders (*Bitis arietans*), pre‐, immediately post‐ and 2 weeks post‐postnatal ecdysis (PNE) in relation to previously published data on adults (Miller et al., 2015). All detectability data are assessed against a random‐choice model (ANOVA One‐factor ANOVA; *F*_4,51_ = 69.31; *p* < .001) and differences (indicated through lettering) are delimited using post hoc Tukey's tests (Error: Between MS = 214.81; df = 51).

Scent‐matching dogs were able to detect pre‐PNE neonate puff adders with 89.1% ± 4.2 accuracy, but detection accuracy of these same neonates immediately post‐PNE and 2 weeks post‐PNE dropped to 56.6% ± 8.0 and 32% ± 6.7, respectively. The detectability difference between pre‐PNE and both post‐PNE treatments is significant, but not between post‐PNE (immediate and 3 weeks). All neonate treatments were significantly more detectable than adults as reported previously (Miller et al., 2015), which were not different from random‐choice (Figure 5; One‐factor ANOVA; *F*_4,51_ = 69.31; *p* < .001). This demonstrates that neonates are not as chemically cryptic as adults.

## 4 DISCUSSION

We found clear phylogenetic patterns in time to PNE, which were associated with several life history traits. This supports our contention that time to PNE is under the selective pressure of several drivers resulting in the reduction and extension of PNE in different species. However, our analysis was limited to a relatively small number of species and only members from the Afrophidia as we were unable to find any measures of time to PNE for any snake species outside this clade. Ancestral state reconstruction revealed that the ancestral time to PNE for the Afrophidia was 11 days, which is modal for our definition of standard PNE (5–17 days). Reduced time to PNE (immediate and early PNE) appears to have evolved in at least four clades independently in our relatively limited data set—particularly within the Viperidae, most Natricinae and some Dipsadinae—and is more prevalent in ambush foraging, viviparous snakes that do not have maternal care. Standard PNE was, by far, the most prevalent condition overall and has generally been retained within the Boidae, Elapidae, Lamprophiidae, Colubrinae and the majority of Crotalinae, and includes all species with maternal care. Furthermore, stochastic mapping indicates that standard PNE is more pliable to either increase or decrease, as opposed to early PNE and extended PNE, which only moves to decrease by one character state. The prevalence of standard PNE supports the contention that it is highly likely the ancestral state for Afrophidia. Extended PNE (>17 days) appears to have evolved in fewer species, and only from within the Colubridae and Pythonidae in our data set. Trained, scent‐matching dogs showed that olfactory detectability of neonate puff adders decreased significantly after PNE and subsequently with time, clearly showing a link between PNE and chemical crypsis. This supports the hypothesis that PNE has an important impact on scent levels of neonates. Together these findings suggest that time to PNE has a significant impact on several aspects of snake biology and is likely to be under strong selection in relation to life history patterns.

Our estimate of 11 days for the ancestral time to PNE in advanced snakes means that there has been subsequent selection for both a reduction and extension in time to PNE in different lineages. In an analysis focussed on maternal care in crotalines, Greene et al. (2002) show that PNE was associated with the termination of care and the dispersal of mother and young from the birthing site. This association between PNE and behaviour is supported by observations of *Python natalensis* (southern African pythons) by Alexander (2018). Greene et al. (2002) suggest that maternal care had likely resulted in the evolution of a longer time to PNE, inferring that a short time to PNE was possibly the ancestral condition since maternal care was an apomorphy. Thus, in their appraisal, time to PNE was under simple directional selection where the presence of maternal care resulted in selection for a lengthening of time to PNE. Their contention is supported by our ancestral reconstruction, which does reveal a marginal increase in time to PNE in crotalines. However, our analysis included a more taxonomically diverse selection of species across the Afrophidia, showing that the ancestral condition for the clade as a whole was only marginally shorter than typical PNE for crotalines.

We found that immediate and early PNE were strongly associated with several life history traits. Intriguingly, ambush foragers were more likely to have early PNE. This significant association was evident despite an obvious and important exception—although crotalines are ambush foragers, the majority have standard PNE. We propose that the crotaline exception is explainable in our framework of competing selective forces and provides further evidence of the link between PNE and chemical crypsis in snakes: Most *Crotalus* and *Sistrurus* species produce an audible rattle in response to approaching danger (Greene, 1997) and thus do not rely on crypsis as a predator avoidance strategy. This behavioural response differs dramatically to the response of the majority of African vipers, particularly *Bitis*, which generally remain immobile and silent in response to approaching danger (Phelps, 2010). For example, unless they happen to be in an exposed situation while moving from one ambush site to another or during mate searching, puff adders will even tolerate being trodden upon without responding if concealed in a lie up (GJA, personal observation). Puff adders are chemically cryptic (Miller, 2022; Miller et al., 2015), whereas the scents of at least two species of rattlesnake, *Crotalus adamanteus* (eastern diamondback rattlesnake) and *C. oreganus helleri* (southern Pacific rattlesnake), have been shown to be easily detected by dogs (Klauber, 1956; Mulholland et al., 2018). Thus, puff adders, and probably several other *Bitis* species appear to rely on extreme crypsis (chemical and visual), while crotalines probably lack chemical crypsis and respond to danger with extravagant warnings. We propose that chemical crypsis has resulted in the selection of early PNE in ambush foragers that rely on crypsis for predator avoidance, but not in ambush foragers that use alternate strategies to avoid predation.

Measures of olfactory detection of neonate puff adders by dogs directly supported our hypothesis that PNE reduces odour of neonates. Postnatal ecdysis typically occurs within hours of birth in puff adders, and our measures showed large decreases in detectability immediately after PNE. Detectability decreased further two weeks later, but remained significantly higher than the detectability of adult *B. arietans*, which are effectively scentless (Miller, 2022; Miller et al., 2015). Thus, complete chemical crypsis appears to take time to manifest in *B. arietans*, suggesting that it may, in part, be dependent on the degree of permeability of the integument, which is reduced over time following birth. Several authors (Agugliaro & Reinert, 2005; Ball, 2004; Graves et al., 1986; Tu et al., 2002) have reported increasing lipid levels in the integument of snakes due to PNE and maturation, and further research may implicate increasing lipid levels in the integument with *B. arietans* chemical crypsis. Mautz (1982) concluded that rates of water loss are three to four times lower in ambushing foragers than in active foragers suggests that this is a potential mechanism for chemical crypsis in some ambushing species. Although the basis of chemical crypsis of *B. arietans* has yet to be elucidated (Miller, 2022), our results do demonstrate that early PNE in puff adders directly improves fitness through an immediate and significant reduction in detectability by macrosmatic predators.

We found that the presence of maternal care was strongly associated with standard PNE. Greene et al. (2002), Schuett et al. (2016), Alexander (2018) and Dwyer et al. (2019) have reported that PNE marks the termination of maternal care and the dispersal of the young and mother from the birthing or nesting site for a range of snake species. Greene et al. (2002) suggest that PNE results in the reduction of the odour that facilitates recognition between mother and offspring, and immediate post‐partum separation effects on kin recognition in *Agkistrodon piscivorus* (cottonmouth, Hoss et al., 2015) appear to support this hypothesis. Only *Sistrurus miliarius* (pygmy rattlesnake) is reported to have maternal care and early PNE (May & Farrell, 2012), but maternal care in this species is brief and does appear to end with PNE (Greene et al., 2002). Patterns within *Crotalus* are also informative—maternal care within the genus is almost ubiquitous (Greene et al., 2002). In our dataset, the only exception to this was *Crotalus durissus* (South American rattlesnake), which has lost maternal care (Greene et al., 2002) and is the only *Crotalus* species known to have immediate PNE. Since maternal care has been shown to provide young with selective advantages in squamates (Greene et al., 2002; O'Connor & Richard, 2004), the presence of maternal care may under some circumstances, preclude the reduction of time to PNE in maternal‐caring species as it would result in premature reduction of the odour necessary for maintaining maternal contact. This link explains the near‐complete absence of a reduction in time to PNE in *Crotalus* and other snakes that have maternal care of young.

A diverse range of species in our dataset have times to PNE that are markedly longer than the ancestral condition. This suggests that in these species, selective forces have operated to extend PNE, even beyond times typical of viviparous, maternal‐caring species. For example, oviparous, egg‐guarding species have significantly longer time to PNE (16 days) than nonguarding, oviparous species (9 days), but it is not apparent what benefit a longer PNE would provide to egg‐guarding species, especially since only a single egg‐guarding snake species (*P. natalensis*) has been shown to exhibit any post hatching maternal care and time to PNE is only slightly greater (13 days) than the ancestral condition in this species (Alexander, 2018). Of the five species that fall into our extended PNE category (>17 days), three are closely related pythons of the *‘Python curtus clade’* and have times ranging between 30 and 60 days. Although extension of time to PNE in egg‐guarding species is most extreme in the Pythonidae, the same pattern holds for each of the families in our data set for which comparisons can be made (Elapidae, Viperidae and Colubridae). Even egg‐eating colubrids, *Dasypeltis medici* and *D. scabra*, which do not egg‐guard, have extended PNE (19–20 days). These small active foragers appear to have little in common with large, ambush foraging and egg‐guarding pythons. Thus, it is not clear what is driving the selection for longer time to PNE or why egg‐guarding would result in increased time to PNE. It is possible that several different selective forces are at play and that the relevant selective forces were not recorded in our study.

Our dataset included only 102 of the >3971 known snake species (Uetz et al., 2022). This limitation resulted from the lack of information for the majority of species. Measures of time to PNE was the primary deficient metric, but in some instances where time to PNE was known, the required gene sequences for the phylogenetic analysis were unavailable. The dearth of published measures of time to PNE is possibly because the importance of this metric has not previously been widely recognised. The low sample size in our analysis is likely to have resulted in an underestimation of the number of times that reduced and extended PNE has arisen, and the inclusion of more species would certainly improve the resolution of these patterns. This would more clearly reveal relationships between PNE and life history traits, possibly clarifying which selective forces have resulted in reduced time to PNE in odorous species, such as *Heterodon*, and extended PNE in egg‐guarding species and *Dasypeltis*. In some instances, the categories that we used to define life history traits were oversimplified. For instance, we used spotted‐blotched body pattern as a proxy for good visual crypsis. Clearly, the plain green coloration of most *Trimeresurus* and *Bothriechis* species make them highly cryptic in their natural arboreal habitat, but they were not scored as such in our simple scheme (even though some *Bothriechis* occur in plain and patterned morphs). Given these shortcomings and room for future improvements, time to PNE has the potential to provide a window into the biology of these species and should therefore be considered a valuable characteristic to include in life history data collection for snakes.

To our knowledge, this is the first study to assess patterns in PNE in detail and relate them to biological traits of snake species. Although previous studies have reported a link between PNE and the termination of mother–neonate bond for several species of snakes, none have explicitly considered time to PNE in an evolutionary context or framework for a large clade, such as Afrophidia. The patterns that we have revealed are useful for generating hypotheses and guiding future research. For example, early PNE may be indicative of chemical crypsis in terrestrial species but is likely to be contraindicative of maternal care of neonates. Although our analysis has not provided an explanation for all the detected patterns, we do believe that it has revealed the interplay of several important selective forces impacting time to PNE and has highlighted some cases that are worthy of further investigation. Standard PNE may have been lost repeatedly within Crotalinae, particularly in tropical viviparous lineages such as *C. durissus* (Greene et al., 2002). This pattern and may be related to maternal attendance, as discussed above, and could be found in other species. For instance, *S. miliarius* (time to PNE = 3.4 days) may be in the process of reducing time to PNE, as seen in *C. durissus*, and is the only species in our study that has maternal care of neonates, albeit only briefly, and early PNE. *Sistrurus miliarius* is also atypical for crotalines in that it generally remains immobile (Farrell et al., 1995) and quiet (Rowe et al., 2002) in response to danger, behaving much like an African *Bitis*. Furthermore, Rowe et al. (2002) conclude that the reduction in rattle size and function in this species is most likely a derived condition. Thus, *S. miliarius* may be a New World example of a species that has adopted crypsis as its primary avoidance strategy to deal with danger and may thus be chemically cryptic, which has resulted in its reduced time to PNE. The brief duration of its maternal care may thus be indicative of a comprise in time to PNE due to competing selective forces. We hope that more studies will include time to PNE to shed light on the selective forces of snake species.

## AUTHOR CONTRIBUTIONS

**Cecilia Wagner:** Conceptualization (equal); data curation (lead); formal analysis (lead); funding acquisition (equal); investigation (lead); methodology (equal); project administration (equal); resources (supporting); software (lead); validation (equal); visualization (equal); writing – original draft (lead); writing – review and editing (lead). **Ashadee K. Miller:** Conceptualization (equal); data curation (supporting); formal analysis (supporting); investigation (supporting); methodology (equal); project administration (supporting); resources (supporting); software (supporting); validation (equal); visualization (equal); writing – original draft (supporting); writing – review and editing (supporting). **Hanlie M. Engelbrecht:** Conceptualization (equal); methodology (equal); software (supporting); validation (equal); writing – original draft (supporting); writing – review and editing (supporting). **Harry W. Greene:** Conceptualization (equal); methodology (equal); validation (equal); writing – review and editing (supporting). **Graham J. Alexander:** Conceptualization (equal); data curation (supporting); funding acquisition (equal); methodology (equal); project administration (equal); resources (lead); supervision (lead); validation (equal); writing – original draft (supporting); writing – review and editing (lead).

## CONFLICT OF INTEREST STATEMENT

The authors have declared that no competing interests exists.

## Supporting information


Data S1:

## Data Availability

The supporting information includes both the time to postnatal ecdysis and life history references from which we sourced the data for all species considered in our analyses (*n* = 102), and is available on Dryad (https://doi.org/10.5061/dryad.zs7h44jfj). Additionally, information on the ancestral average days to postnatal ecdysis of the clades of interest, based on the ancestral state reconstruction analysis, is available in the Appendix 1 section. Data supporting the results in this paper are available in the Appendix 1 and Data S1.

## APPENDIX 1

**TABLE A1:** Ancestral average days to postnatal ecdysis of the clades of interest based on the ancestral state reconstruction analysis of 102 Afrophidia species (10 Henophidia + 92 Caenophidia).

Clade	Ancestral average days to postnatal ecdysis
Ancestral	11.0
Henophidia	12.2
Boidae	11.5
Pythonidae	20.3
Caenophidia	8.5
Colubridae	8.5
Colubrinae	10.3
*Dasypeltis*	16.0
Dipsadinae	5.8
*Heterodon*	0.9
Natricinae	5.0
*Clonophis*, *Thamnophis*, *Nerodia*	2.2
Elapidae	
Elapinae, Hydrophiinae	10.9
Lamprophiidae	
Pseudoxyrhophiinae	9.8
Lamprophiinae	11.4
Viperidae	5.7
Crotalinae	7.1
*Atropoides*	4.3
Viperinae	4.6
*Bitis*	2.4
