# Supplementary material for: When to shed? Patterns and drivers of time to first ecdysis in snakes
Source: Ecol Evol. 2023 Aug 2;13(8):e10364. doi: 10.1002/ece3.10364 (PMC10396665; doi:10.1002/ece3.10364)
Supplement: Supplementary file 1 — Data S1: [file ECE3-13-e10364-s001.docx]

**When to shed? Patterns and drivers of time to first ecdysis in snakes – Supporting Information**

Cecilia Wagner^1^, Ashadee K. Miller^1^, Hanlie M. Engelbrecht^1^, Harry W. Greene^2^ and Graham J. Alexander^1*^

^1^School of Animal, Plant and Environmental Sciences, University of the Witwatersrand, Johannesburg, South Africa

^2^Department of Ecology and Evolutionary Biology, Corson Hall, Cornell University, Ithaca, New York 14853, USA

**Correspondence**

Graham J. Alexander, School of Animal, Plant and Environmental Sciences, University of the Witwatersrand, 1 Jan Smuts Ave, Braamfontein, Johannesburg, 2000, South Africa

E-mail: [graham.alexander@wits.ac.za](mailto:graham.alexander@wits.ac.za)

# **SUPPORTING INFORMATION**

**S1 Table.** Time to postnatal ecdysis and the reference(s) it is based on per sampled species. Average days to postnatal ecdysis (PNE) and references of records on time to PNE of all sampled species (n = 102). * = Personal communication, ** = Institution or group, *** = as referenced by https://animaldiversity.org

| Species Name | Average PNE | Reference |
| --- | --- | --- |
| *Agkistrodon bilineatus* | 9.0 | (Dwyer et al., 2019) |
| *Agkistrodon contortrix* | 7.8 | (Schuett, 1982; Boerema, 1991; Gross, 2017) |
| *Agkistrodon piscivorus* | 11.0 | (Wharton, 1960; Hoss, 2013; Hoss & Clark, 2014) |
| *Antaresia stimsoni* | 12.0 | Ssnakess Forum (https://www.ssnakess.com/forums/)* |
| *Atropoides nummifer* | 0.5 | (Greene et al., 2002) |
| *Atropoides picadoi* | 0.5 | (Greene et al., 2002) |
| *Bitis arietans* | 0.5 | AKM Unpublished Data |
| *Bitis caudalis* | 0.5 | Roberts, Anton* |
| *Bitis gabonica* | 0.5 | (Akester, 1984); Roberts, Anton* |
| *Bitis peringueyi* | 0.5 | (Robinson & Hughes, 1978) |
| *Boa constrictor* | 9.7 | Krezos, Harrison (Prime Predators) *; ReptileForum* |
| *Boaedon capensis* | 12.0 | GJA Unpublished Data |
| *Bothriechis lateralis* | 0.5 | (Greene et al., 2002) |
| *Bothriechis schlegelii* | 6.0 | (Greene et al., 2002) |
| *Bothrops alternatus* | 12.0 | (Pezzano, 2002) |
| *Bothrops asper* | 14.5 | (Solórzano & Cerdas, 1989) |
| *Bothrops atrox* | 11.5 | Vermeulen, Blake (Viper Brothers)* |
| *Bothrops jararaca* | 1.5 | (Breno et al., 1990) |
| *Bungarus fasciatus* | 12.5 | (Chanhome et al., 2001) |
| *Calloselasma rhodostoma* | 11.3 | (Chanhome et al., 2001) |
| *Causus rhombeatus* | 8.0 | Rowberry, Caleb* |
| *Cerastes cerastes* | 5.0 | (Peet, 1992) |
| *Chilabothrus subflavus* | 8.3 | Toronto Zoo (Species360) ** |
| *Clonophis kirtlandii* | 2.0 | (Powell & Parmerlee, 1991) |
| *Coelognathus helena* | 7.0 | (Mehta, 2009) |
| *Corallus caninus* | 16.7 | (Murphy et al., 1978); Toronto Zoo (Species360) ** |
| *Coronella austriaca* | 7.0 | (Pernetta et al., 2009) |
| *Crotalus adamanteus* | 12.5 | (Butler et al., 1995; Ernst & Ernst, 2012***; Wray et al., 2015) |
| *Crotalus atrox* | 6.5 | (Price, 1988; Schuett et al., 2013, 2016) |
| *Crotalus cerastes* | 10.0 | (Reiserer & Schuett, 2008; Reiserer et al., 2008) |
| *Crotalus durissus* | 0.5 | (Greene et al., 2002) |
| *Crotalus horridus* | 9.2 | (Beaupre & Zaidan, 2001; Ernst & Ernst, 2003***; Brown et al., 2007; Howze et al., 2012; Wray et al., 2015) |
| *Crotalus lepidus* | 9.0 | (Kauffeld, 1943) |
| *Crotalus molossus* | 9.5 | (Greene et al., 2002) |
| *Crotalus polystictus* | 11.0 | (Setser et al., 2010) |
| *Crotalus pricei* | 7.0 | (Kauffeld, 1943) |
| *Crotalus viridis* | 12.9 | (Cunningham, 1959; Greene et al., 2002; Holycross & Fawcett, 2002) |
| *Daboia russelii* | 0.5 | (Chanhome et al., 2001) |
| *Dasypeltis inornata* | 14.0 | Rowberry, Caleb* |
| *Dasypeltis medici* | 20.0 | (Paterna, 2017) |
| *Dasypeltis scabra* | 19.0 | Barends, Jody Michael* |
| *Dendroaspis angusticeps* | 11.8 | (Haagner & Morgan, 1989); Rowberry, Caleb* |
| *Dendroaspis polylepis* | 13.3 | (Haagner & Morgan, 1993); Rowberry, Caleb* |
| *Dispholidus typus* | 10.3 | (Boycott, 1990)***; Rowberry, Caleb* |
| *Elaphe dione* | 7.5 | (Popovskaya, 2001; Dujsebayeva, 2008) |
| *Elaphe schrenckii* | 9.0 | (Langeveld, 1994) |
| *Gonyosoma boulengeri* | 10.0 | (Kane et al., 2017) |
| *Gonyosoma oxycephalum* | 7.0 | (Even, 1989); Toronto Zoo (Species360)** |
| *Heterodon kennerlyi* | 0.5 | (Hay, 1892); ReptileForum (https://www.reptileforums.co.uk/)* |
| *Heterodon nasicus* | 0.5 | (Hay, 1892); ReptileForum (https://www.reptileforums.co.uk/)* |
| *Hypnale hypnale* | 8.5 | (De Silva & Toriba, 1984; Greene et al., 2002) |
| *Lachesis melanocephala* | 11.9 | Baldwin, Brett*; (De Plecker & Dwyer, 2020) |
| *Lachesis muta* | 23.5 | (Boyer et al., 1989); Recchio, Ian* |
| *Lachesis stenophrys* | 15.0 | (Greene et al., 2002) |
| *Lampropeltis calligaster* | 8.5 | (Tryon & Carl, 1980) |
| *Lampropeltis getula* | 8.8 | (Ernst & Barbour, 1989***; Tu et al., 2002) |
| *Lampropeltis triangulum* | 9.7 | (Herman, 1979; Tryon & Murphy, 1982; Hammack, 1989) |
| *Lamprophis aurora* | 12.0 | Alexander Herp Lab** |
| *Langaha madagascariensis* | 10.0 | (Krysko, 2003) |
| *Lycodon ruhstrati* | 6.5 | (Norval et al., 2007) |
| *Micrurus fulvius* | 14.0 | (Campbell, 1973) |
| *Montivipera latifii* | 6.0 | (Kian et al., 2011) |
| *Naja annulifera* | 9.0 | Alexander Herp Lab**; Rowberry, Caleb* |
| *Naja kaouthia* | 8.0 | (Chanhome et al., 2001) |
| *Naja melanoleuca* | 8.5 | (Tryon, 1979) |
| *Naja nigricollis* | 14.5 | (Haagner & Carpenter, 1992) |
| *Naja siamensis* | 12.5 | (Chanhome et al., 2001) |
| *Natrix natrix* | 8.0 | (Alibardi, 2002) |
| *Nerodia fasciata* | 0.5 | (Slejipen, 1991) |
| *Nerodia taxispilota* | 1.0 | (Franklin, 1944; Mills, 2002) |
| *Ophiophagus hannah* | 9.5 | (Chanhome et al., 2001) |
| *Oreocryptophis porphyraceus* | 7.5 | (Romer, 1979) |
| *Oxybelis wilsoni* | 13.0 | (Groves, 1995) |
| *Pantherophis guttatus* | 7.8 | (Griswold, 2001); ReptileForum (https://www.reptileforums.co.uk/)* |
| *Pantherophis obsoletus* | 6.5 | (Termeer, 1991) |
| *Philothamnus semivariegatus* | 12.0 | Alexander Herp Lab** |
| *Pituophis catenifer* | 11.0 | (Piriou et al., 2015) |
| *Pituophis melanoleucus* | 11.3 | (Cliburn, 1976); Lee 1967 in (Petzold, 1967) |
| *Proatheris superciliaris* | 0.5 | Roberts, Anton* |
| *Protobothrops cornutus* | 9.0 | (Shiryaev et al., 2007) |
| *Protobothrops jerdonii* | 0.5 | (Orlov et al., 2002) |
| *Protobothrops mangshanensis* | 14.0 | Gumprecht et al. (2002) in (http://ielc.libguides.com/sdzg/factsheets/mangshanpitviper/reproduction, accessed: 18 Dec 2019) |
| *Protobothrops maolanensis* | 8.5 | (Kropachev et al., 2015) |
| *Protobothrops sieversorum* | 10.0 | (Shiryaev et al., 2007) |
| *Pseudonaja textilis* | 8.0 | (Alibardi, 2002) |
| *Python breitensteini* | 45.0 | ReptileForum (https://www.reptileforums.co.uk/)* |
| *Python brongersmai* | 45.0 | (Ball, 2004); ReptileForum (https://www.reptileforums.co.uk/)* |
| *Python curtus* | 45.0 | (Ball, 2004); ReptileForum (https://www.reptileforums.co.uk/)* |
| *Python molurus* | 12.2 | (Walsh & Murphy, 2003) |
| *Python natalensis* | 13.0 | (Alexander, 2018) |
| *Python regius* | 8.5 | Mezsar, Calvin* |
| *Sistrurus catenatus* | 6.1 | Toronto Zoo (Species360)**; Warner, Jon*; (Schuett et al., 1984; Wastell & Mackessy, 2016) |
| *Sistrurus miliarius* | 3.4 | (May & Farrell, 2012); May & Farrell Unpublished Data |
| *Spilotes sulphureus* | 11.0 | (Valencia & Garzon-Tello, 2018) |
| *Thamnophis radix* | 0.5 | (Platvoet, 1991) |
| *Tretanorhinus variabilis* | 7.0 | (Petzold, 1967) |
| *Trimeresurus albolabris* | 2.3 | (Drent, 1991; Orlov et al., 2002; Orlov et al., 2002) |
| *Trimeresurus macrops* | 0.5 | (https://www.hetterrarium.com/en_GB/a-47972164/venomous-reptiles/trimeresurus-macrops-info-care/, accessed 18 Dec 2019) |
| *Trimeresurus puniceus* | 12.5 | Roberts, Anton*; (Orlov et al., 2002) |
| *Vipera ammodytes* | 0.5 | Vermeulen, Marcel (Viper Brothers)* |
| *Vipera berus* | 1.5 | (Smith, 1951; Street, 1979; Greene et al., 2002) |
| *Vipera kaznakovi* | 2.0 | (Göçmen et al., 2013) |

**S2 Table.** References of life history traits of sampled species (n = 102). Life history traits included family, subfamily, and body pattern, foraging mode, parity mode, maternal care, and biogeographic region.

| Species Name | Animal  Diversity | IUCN  Red List | Reptile  Database | Wikipedia | Literature | Other |
| --- | --- | --- | --- | --- | --- | --- |
| *Agkistrodon bilineatus* |  | x | x | x | (Dwyer et al., 2019) |  |
| *Agkistrodon contortrix* | x | x | x |  |  |  |
| *Agkistrodon piscivorus* | x | x | x |  |  |  |
| *Antaresia stimsoni* | x | x |  | x |  |  |
| *Atropoides nummifer* |  | x | x |  |  |  |
| *Atropoides picadoi* |  | x | x |  |  |  |
| *Bitis arietans* | x |  |  |  |  |  |
| *Bitis caudalis* |  |  | x | x | (Marais, 2011) |  |
| *Bitis gabonica* | x |  |  |  |  |  |
| *Bitis peringueyi* |  | x | x | x | (Robinson & Hughes, 1978) |  |
| *Boa constrictor* | x |  |  |  |  |  |
| *Boaedon capensis* |  |  |  | x |  |  |
| *Bothriechis lateralis* |  | x |  | x |  |  |
| *Bothriechis schlegelii* |  | x |  | x |  |  |
| *Bothrops alternatus* |  |  |  | x | (Pezzano, 2002) |  |
| *Bothrops asper* | x |  |  |  |  |  |
| *Bothrops atrox* | x |  |  |  |  |  |
| *Bothrops jararaca* | x | x |  |  |  |  |
| *Bungarus fasciatus* |  |  |  | x | (Chanhome et al., 2001) |  |
| *Calloselasma rhodostoma* |  | x |  | x |  |  |
| *Causus rhombeatus* |  |  |  | x | (Marais, 2011) |  |
| *Cerastes cerastes* | x | x |  |  |  |  |
| *Chilabothrus subflavus* |  |  |  | x |  | Newman, Brett pers. com. |
| *Clonophis kirtlandii* |  | x |  | x |  | www.biokids.umich.edu |
| *Coelognathus helena* |  |  |  | x |  | www.hetterrarium.com |
| *Corallus caninus* | x |  |  |  |  |  |
| *Coronella austriaca* |  | x |  |  | (Pernetta et al., 2009) |  |
| *Crotalus adamanteus* | x |  |  |  |  |  |
| *Crotalus atrox* | x | x |  |  |  |  |
| *Crotalus cerastes* | x |  |  |  |  |  |
| *Crotalus durissus* |  | x |  | x | (Greene et al., 2002) |  |
| *Crotalus horridus* | x | x |  | x |  |  |
| *Crotalus lepidus* |  | x |  | x |  |  |
| *Crotalus molossus* | x | x |  |  |  |  |
| *Crotalus polystictus* |  | x |  |  | (Setser et al., 2010) |  |
| *Crotalus pricei* |  | x |  |  |  |  |
| *Crotalus viridis* | x | x |  | x |  |  |
| *Daboia russelii* |  | x |  | x |  |  |
| *Dasypeltis inornata* |  | x |  | x |  |  |
| *Dasypeltis medici* |  |  |  | x | (Paterna, 2017) |  |
| *Dasypeltis scabra* |  | x |  | x |  |  |
| *Dendroaspis angusticeps* |  |  |  | x | (Haagner & Morgan, 1989) |  |
| *Dendroaspis polylepis* | x | x |  |  | (Haagner & Morgan, 1993) |  |
| *Dispholidus typus* | x |  |  |  | (Marais, 2011) |  |
| *Elaphe dione* |  | x | x |  |  | www.hetterrarium.com |
| *Elaphe schrenckii* |  |  |  | x |  | www.livescience.com |
| *Gonyosoma boulengeri* |  |  |  | x |  |  |
| *Gonyosoma oxycephalum* |  |  |  | x |  |  |
| *Heterodon kennerlyi* |  |  | x | x |  |  |
| *Heterodon nasicus* | x | x |  | x |  |  |
| *Hypnale hypnale* |  |  |  | x |  |  |
| *Lachesis melanocephala* |  |  | x |  | (De Plecker & Dwyer, 2020) |  |
| *Lachesis muta* | x |  |  | x |  |  |
| *Lachesis stenophrys* |  |  |  | x |  |  |
| *Lampropeltis calligaster* |  | x |  | x | (Tryon & Carl, 1980) |  |
| *Lampropeltis getula* | x | x |  |  |  |  |
| *Lampropeltis triangulum* | x | x |  |  |  |  |
| *Lamprophis aurora* |  | x |  | x |  |  |
| *Langaha madagascariensis* |  | x |  | x |  |  |
| *Lycodon ruhstrati* |  | x |  | x | (Norval et al., 2007) |  |
| *Micrurus fulvius* | x | x |  | x | (Campbell, 1973) |  |
| *Montivipera latifii* |  | x |  |  | (Kian et al., 2011) |  |
| *Naja annulifera* |  |  |  | x | (Marais, 2011) |  |
| *Naja kaouthia* |  |  |  | x |  |  |
| *Naja melanoleuca* | x |  |  | x | (Marais, 2011) |  |
| *Naja nigricollis* |  |  |  | x |  |  |
| *Naja siamensis* |  | x |  | x | (Chanhome et al., 2001) |  |
| *Natrix natrix* |  |  |  | x |  |  |
| *Nerodia fasciata* | x | x |  |  |  |  |
| *Nerodia taxispilota* | x | x |  |  |  |  |
| *Ophiophagus hannah* | x | x |  |  |  |  |
| *Oreocryptophis porphyraceus* |  |  |  | x | (Romer, 1979) |  |
| *Oxybelis wilsoni* |  | x |  |  | (Groves, 1995) |  |
| *Pantherophis guttatus* | x | x |  | x |  |  |
| *Pantherophis obsoletus* | x | x |  |  |  |  |
| *Philothamnus semivariegatus* |  |  |  | x | (Marais, 2011) |  |
| *Pituophis catenifer* | x | x |  |  |  |  |
| *Pituophis melanoleucus* | x | x |  |  |  |  |
| *Proatheris superciliaris* |  |  |  | x |  |  |
| *Protobothrops cornutus* |  | x |  |  | (Shiryaev et al., 2007) |  |
| *Protobothrops jerdonii* |  | x |  |  | (Orlov et al., 2002) |  |
| *Protobothrops mangshanensis* |  | x |  | x |  |  |
| *Protobothrops maolanensis* |  | x |  |  |  |  |
| *Protobothrops sieversorum* |  | x |  |  | (Shiryaev et al., 2007) |  |
| *Pseudonaja textilis* |  | x |  | x |  |  |
| *Python breitensteini* |  | x |  |  |  |  |
| *Python curtus* |  | x |  | x |  |  |
| *Python molurus* |  | x |  | x |  |  |
| *Python natalensis* | x |  |  |  |  |  |
| *Python regius* |  |  |  | x |  |  |
| *Python brongersmai* | x | x |  |  |  |  |
| *Sistrurus catenatus* | x | x |  |  |  |  |
| *Sistrurus miliarius* | x | x |  |  | (May & Farrell, 2012) |  |
| *Spilotes sulphureus* |  |  |  |  | (Valencia & Garzon-Tello, 2018) |  |
| *Thamnophis radix* | x | x |  |  |  |  |
| *Tretanorhinus variabilis* |  | x |  |  | (Petzold, 1967) |  |
| *Trimeresurus albolabris* |  | x |  |  | (Orlov et al., 2002) |  |
| *Trimeresurus macrops* |  | x |  |  |  |  |
| *Trimeresurus puniceus* |  | x |  |  | (Orlov et al., 2002) |  |
| *Vipera ammodytes* |  | x |  |  |  |  |
| *Vipera berus* | x |  |  | x |  |  |
| *Vipera kaznakovi* |  | x |  |  | (Göçmen et al., 2013) |  |

# **SUPPORTING INFORMATION REFERENCES**

Akester, J. (1984). Further observations on the breeding of the Gaboon viper (*Bitis g. gabonica*) in captivity (Serpentes: Viperidae). *Arnoldia Zimbabwe*, 9(13), 217–222.

Alexander, G.J. (2018). Reproductive biology and maternal care of neonates in southern African python (*Python natalensis*). *Journal of Zoology*, 305(London), 141–148.

Alibardi, L. (2002). Ultrastructure of the embryonic snake skin and putative role of histidine in the differentiation of the shedding complex. *Journal of Morphology*, 251(2), 149–168. https://doi.org/10.1002/jmor.1080.

Ball, J.C. (2004). The first shed skin of neonate corn snakes is chemically different from adult shed skins. *Journal of Herpetology*, 38(1), 124–127. https://doi.org/10.1670/96-03n.

Beaupre, S.J. & Zaidan, F. (2001). Scaling of CO2 production in the timber rattlesnake (*Crotalus horridus*), with comments on cost of growth in neonates and comparative patterns. *Physiological and Biochemical Zoology*, 74(5), 757–768. https://doi.org/10.1086/322965.

Boerema, H. (1991). Breeding Results: *Agkistrodon contortrix mokeson* – Copperhead. *Litteratura Serpentium*, 11(5), 114.

Boycott, R.D. (1990). Observations on reproduction in southern African Boomslang. *Journal of the Herpetological Association of Africa*, 38(1), 51–52.

Boyer, D.M., Mitchell, L.A. & Murphy, J.B. (1989). Reproduction and husbandry of the Bushmaster *Lachesis m. muta* at the Dallas Zoo. *International Zoo Yearbook*, 28(1), 190–194. https://doi.org/10.1111/j.1748-1090.1989.tb03279.x.

Breno, M.C., Yamanouye, N., Prezoto, B.C., Lazari, M.F.M., Toffoletto, O., & Picarelli, Z.P*.* (1990). Maintenance of the snake *Bothrops jararaca* (Wied, 1824) in captivity. *Snake*, 22(2), 126–130.

Brown, W.S., Kéry, M. & Hines, J.E. (2007). Survival of timber rattlesnakes (*Crotalus horridus*) estimated by capture-recapture models in relation to age, sex, color morph, time, and birthplace. *Copeia*, 2007(3), 656–671. https://doi.org/10.1643/0045-8511(2007)2007[656:SOTRCH]2.0.CO;2.

Butler, J.A., Hull, T.W. & Franz, R. (1995). Neonate aggregations and maternal attendance of young in the eastern diamondback rattlesnake, *Crotalus adamanteus*. *Copeia*, 1995, 196–198.

Campbell, J.A. (1973). A captive hatching of *Micrurus fulvius tenere* (Serpentes, Elapidae). *Journal of Herpetology*, 7(3), 312–315.

Chanhome, L., Jintakune, P., Wilde, H., & Cox, M.J*.* (2001). Venomous snake husbandry in Thailand. *Wilderness and Environmental Medicine*, 12(1), 17–23. https://doi.org/10.1580/1080-6032(2001)012[0017:VSHIT]2.0.CO;2.

Cliburn, J.W. (1976). Observations of ecdysis in the black pine snake, *Pituophis melanoleucus lodingi* (Reptilia, Serpentes, Colubridae). *Journal of Herpetology*, 10(4), 299–301.

Cunningham, J.D. (1959). Reproduction and food of some California snakes. *Herpetologica*, 15(1), 17–19.

Drent, J. (1991). Breeding Results: *Trimeresurus albolabris* (F2). *Litteratura Serpentium*, 11(5), 115.

Dujsebayeva, T.N. (2008). The skin development in the Pallas’ Coluber, *Elaphe dione* (Pallas, 1773) (Serpentes, Colubridae). *Russian Journal of Herpetology*, 15(1), 44–54.

Dwyer, Q., Schuett, G.W. & Greene, H.W. (2019). *Agkistrodon cf. bilineatus* (Northern Cantil). Maternal care. *Herpetological Review*, 50(4), 741–742.

Ernst, C. & Barbour, R. (1989). *Snakes of Eastern North America*. Virginia: George Mason University Press.

Ernst, C. & Ernst, E. (2003). *Snakes of the United States and Canada*. Washington, D.C.: Smithsonian Books.

Ernst, C. & Ernst, E. (2012). *Venomous Reptiles of the United States, Canada, and Northern Mexico*. Volume 2. Baltimore, MD: The John Hopkins University Press.

Even, E. (1989). Care and repeated breeding of the green ratsnake (*Gonyosoma oxycephala*). *Litteratura Serpentium English Edition*, 9(4), 145–155.

Franklin, M.A. (1944). Notes on the young of the brown water snake. *Copeia*, 4(250).

Göçmen, B., Çiçek, K., Iğci, N., & Akman, B*.* (2013). Data on the reproduction of a Caucasian Viper, *Vipera kaznakovi,* Nikolsky, 1909 (Serpentes: Viperidae) from Hopa (Northeastern Anatolia, Turkey). *Ecologia Balkanica*, 5(2), 99–101.

Greene, H.W., May, P.G., Hardy, D.L.S., Sciturro, J.M., & Farrell, T.M*.* (2002). Parental behaviour by vipers. In G.W. Schuett, M. Hoggren, M.E. Douglas, & H.W. Greene (Eds.), *Biology of Vipers*. Eagle Mountain Publishing, pp. 179–205.

Griswold, W.G. (2001). Captive care and breeding of the corn snake, *Elaphe guttata*. *Journal of Herpetological Medicine and Surgery*, 11(4), 35–40. https://doi.org/10.5818/1529-9651.11.4.35.

Gross, I.P. (2017). *Habitat use, dispersal, and hibernation of maternal and neonatal copperheads (Crotalinae: Agkistrodon) in a managed southeastern forest*. MSc thesis. Alabama A&M University, Normal.

Groves, J.D. (1995). Reproduction and feeding behavior of *Oxybelis wilsoni*, a new species of vine snake (Serpentes: Colubridae). *Revista de biología tropical*, 43(1–3), 307–309.

Haagner, G. V. & Morgan, D.R. (1989). The captive propagation of the eastern green mamba *Dendroaspis angusticeps*. *International Zoo Yearbook*, 28(1), 195–199. https://doi.org/10.1111/j.1748-1090.1989.tb03280.x.

Haagner, G. V. & Morgan, D.R. (1993). The maintenance and propagation of the black mamba *Dendroaspis polylepis* at the Manyeleti Reptile Centre, Eastern Transvaal. *International Zoo Yearbook*, 32(1), 191–196. https://doi.org/10.1111/j.1748-1090.1993.tb03534.x.

Haagner, G.V. & Carpenter, G. (1992). Notes on the captive breeding of the black spitting cobra, *Naja nigricollis woodi* (Serpentes: Elapidae). *The Journal of the Herpetological Association of Africa*, 41(1), 22–24.

Hammack, S.H. (1989). Reproduction of the Colombian milk snake *Lampropeltis triangulum andesiana* at the Dallas Zoo. *International Zoo Yearbook*, 28(1), 172–177. https://doi.org/10.1111/j.1748-1090.1989.tb03276.x.

Hay, O.P. (1892). On the breeding habits, eggs, and young of certain snakes. *Proceedings of the United States National Museum*, XV, 385–397.

Herman, D.W. (1979). Breeding the Jaliscan milk snake: *Lampropeltis triangulum arcifera* [Plate 25]: at Atlanta Zoo. *International Zoo Yearbook*, 19(1), 96–97.

Holycross, A.T. & Fawcett, J.D. (2002). Observations on neonatal aggregations and associated behaviors in the prairie rattlesnake, *Crotalus viridis viridis*. *American Midland Naturalist*, 148(1), 181–184. https://doi.org/10.1674/0003-0031(2002)148[0181:OONAAA]2.0.CO;2.

Hoss, S.K. (2013). *Maternal attendance of young in cottonmouths (Agkistrodon piscivorous): Adaptive value and hormonal mechanisms*. PhD thesis. University of California & San Diego State University.

Hoss, S.K. & Clark, R.W. (2014). Mother cottonmouths (*Agkistrodon piscivorus*) alter their antipredator behavior in the presence of neonates. *Ethology*, 120(9), 933–941. https://doi.org/10.1111/eth.12265.

Howze, J.M., Stohlgren, K.M., Schlimm, E.M., & Smith, L.L. (2012). Dispersal of neonate timber rattlesnakes (*Crotalus horridus*) in the southeastern coastal plain. *Journal of Herpetology*, 46(3), 417–422. https://doi.org/10.1670/10-126.

Kane, D., Gill, I., Harding, L., Capon, J., Franklin, M., Servini, F., Tapley, B., & Michaels, C.J. (2017). Captive husbandry and breeding of *Gonyosoma boulengeri*. *Herpetological Bulletin*, (139), 7–11.

Kauffeld, C.F. (1943). Growth and feeding of newborn Price’s and Green Rock Rattlesnakes. *American Midland Naturalist*, 29(3), 607. https://doi.org/10.2307/2421150.

Kian, N., Kaboli, M., Karami, M., Alizadeh, A., Teymurzadeh, S., Khalilbeigi, N., Murphy, J.B., & Nourani, E*.* (2011). Captive management and reproductive biology of Latifi’s viper (*Montivipera latifii*) (Squamata: Viperidae) at Razi Institute and Tehran University in Iran. *Herpetological Review*, 42(4), 535–539.

Kropachev, I.I., Shiryaev, K.A., Thien, N.T., & Orlov, N.L*.* (2015). New record of *Protobothrops cf. maolanensis* in northeastern Vietnam, with data on its morphology and biology. *Russian Journal of Herpetology*, 22(2), 93–102.

Krysko, K.L. (2003). Reproduction in the Madagascar leaf-nosed snake*, Langaha madagascariensis* (Serpentes: Colubridae: Pseudoxyrhophiinae). *Journal of the Herpetological Association of Africa*, 52(1), 61–68. https://doi.org/10.1080/21564574.2003.9635478.

Langeveld, C.M. (1994). The captive care and breeding of the Amur ratsnake, *Elaphe schrencki schrencki* (Strauch 1873) and the southern Amur ratsnake, *Elaphe schrencki anomala* (Boulenger 1916). *Litteratura Serpentium*, 14(5), 134–141.

Marais, J. (2011). *A Complete Guide to the Snakes of Southern Africa*. 2nd edition. Penguin Random House.

May, P.G. & Farrell, T.M. (2012). Growth patterns of dusky pygmy rattlesnakes (*Sistrurus miliarius barbouri*) from Central Florida. *Herpetological Monographs*, 26(26), 58–79. https://doi.org/10.1655/HERPMONOGRAPHS-D-11-00003.1.

Mehta, R.S. (2009). Early experience shapes the development of behavioral repertoires of hatchling snakes. *Journal of Ethology*, 27(1), 143–151. https://doi.org/10.1007/s10164-008-0097-9.

Mills, M.S. (2002). *Ecology and life history of the brown watersnake (Nerodia taxispilota)*. PhD thesis. The University of Georgia, Athens.

Murphy, J.B., Barker, D.G. & Tryon, B.W. (1978). Miscellaneous notes on the reproductive biology of reptiles. 2. Eleven species of the family Boidae, Genera *Candoia, Corallus, Epicrates* and *Python*. *Journal of Herpetology*, 12(3), 385–390.

Norval, G., Mao, J.J. & Chu, H.P. (2007). Oviposition and early growth of unfed neonates of the mountain wolf snake *Lycodon ruhstrati ruhstrati* (Squamata: Colubridae). *Current Herpetology*, 26(1), 49–51. https://doi.org/10.5358/hsj.26.49.

Orlov, N., Natalia, A., Barabanov, A., Ryabov, S., & Khalikov, R*.* (2002). Diversity of vipers (Azemiopinae, Crotalinae ) in East, Southeast, and South Asia: Annotated checklist and natural history data (Reptilia: Squamata: Serpentes: Viperidae). *Faunistische Abhandlungen des Staatlichen Museums für Tierkunde Dresden*, 23(812), 177–218.

Orlov, N., Ananjeva, N. & Khalikov, R. (2002). Natural history of pitvipers in Eastern and Southeastern Asia. *Biology of the Vipers*, 345–360.

Paterna, A. (2017). Reproduction cycle of the eastern egg-eater snake *Dasypeltis medici medici* (Bianconi, 1859) in captivity. *Russian Journal of Herpetology*, 24(3), 228–234. https://doi.org/10.30906/1026-2296-2019-24-3-228-234.

Peet, Smetsers. (1992). Keeping and breeding *Cerastes cerastes karlhartli*, the horned viper. *Litteratura Serpentium*, 12(4), 93–96. https://doi.org/10.2307/1446776.

Pernetta, A.P., Reading, C.J. & Allen, J.A. (2009). Chemoreception and kin discrimination by neonate smooth snakes, *Coronella austriaca*. *Animal Behaviour*, 77(2), 363–368. https://doi.org/10.1016/j.anbehav.2008.10.008.

Petzold, H.-G. (1967). Some remarks on the breeding biology and the keeping of *Tretanorhinus variabilis*, a water snake of Cuba. *Herpetologica*, 23(3), 242–246.

Pezzano, V. (2002). Reproduction of *Bothrops alternatus* (Dumeril, Bibron & Dumeril, 1854) in captivity. *Journal of Fish Biology*, 61, 196–206.

Piriou, A., Renard, M., Norval, G., Cooper, J.E., & Mao, J.J*.* (2015). A description of an excrescence in a captive-bred specimen of *Pituophis catenifer sayi* (Serpentes, Colubridae). *Herpetology Notes*, 8(October), 507–509.

Platvoet, H.-J. (1991). Breeding Results: *Thamnophis radix haydeni*. *Litteratura Serpentium*, 11(5), 114.

De Plecker, R. & Dwyer, Q. (2020). First breeding of the black-headed bushmaster (*Lachesis melanocephala*) in Costa Rica. *Herpetological Review*, 51(1), 57–64.

Popovskaya, S.P. (2001). *First shedding in the different snake species of the family Colubridae*, *Problems in Herpetology*. Moscow.

Powell, R. & Parmerlee, J.S.Jr. (1991). Notes on reproduction in *Clonophis kirtlandii* (Serpentes: Colubridae). *Bulletin of the Chicago Herpetological Society*, 26(2), 32.

Price, A.H. (1988). Observations on maternal behavior and neonate aggregation in the western diamondback rattlesnake, *Crotalus atrox* (Crotalidae). *The Southwestern Naturalist*, 33(3), 370–373.

Reiserer, R.S. & Schuett, G.W. (2008). Aggressive mimicry in neonates of the sidewinder rattlesnake, *Crotalus cerastes* (Serpentes: Viperidae): Stimulus control and visual perception of prey luring. *Biological Journal of the Linnean Society*, 95(1), 81–91. https://doi.org/10.1111/j.1095-8312.2008.01016.x.

Reiserer, R.S., Schuett, G.W. & Earley, R.L. (2008). Dynamic aggregations of newborn sibling rattlesnakes exhibit stable thermoregulatory properties. *Journal of Zoology*, 274(3), 277–283. https://doi.org/10.1111/j.1469-7998.2007.00383.x.

Robinson, M.D. & Hughes, D.A. (1978). Observations on the natural history of Peringuey’s Adder, *Bitis peringueyi* (Boulenger) (Reptilia: Viperidae). *Annals of the Transvaal Museum*, 31(November 1971), 189–196.

Romer, J.D. (1979). Captive care and breeding of a little known Chinese snake: *Elaphe porphyracea nigrofasciata*. *International Zoo Yearbook*, 19(1), 92–94. https://doi.org/10.1111/j.1748-1090.1979.tb00537.x.

Schuett, G.W. (1982). A copperhead (*Agkistrodon contortrix*) brood produced from autumn copulations. *Copeia*, 1982(3), 700. https://doi.org/10.2307/1444673.

Schuett, G.W., Repp, R.A., Hoss, S.K., & Herrmann, H.W*.* (2013). Environmentally cued parturition in a desert rattlesnake, *Crotalus atrox*. *Biological Journal of the Linnean Society*, 110(4), 866–877. https://doi.org/10.1111/bij.12166.

Schuett, G.W., Clark, R.W., Repp, R.A., Amarello, M., & Greene, H.W*.* (2016). Social behavior of rattlesnakes: A shifting paradigm. in G.W. Schuett, M.J. Feldner, C.F. Smith, & R.S. Reiserer (Eds.). *Rattlesnakes of Arizona*. 2nd edition. ECO Wear & Publishing, pp. 161–242.

Schuett, G.W., Clark, D.L. & Kraus, F. (1984). Feeding mimicry in the rattlesnake *Sistrurus catenatus*, with comments on the evolution of the rattle. *Animal Behaviour*, 32(2), 625–626.

Setser, K. *et al.* (2010) ‘Reproductive ecology of female Mexican lance-headed rattlesnakes’, *Journal of Zoology*, 281(3), pp. 175–182. Available at: https://doi.org/10.1111/j.1469-7998.2010.00692.x.

Shiryaev, K.A., Mociño-Deloya, E., Pleguezuelos, J.M., Lazcano, D., & Kardon, A*.* (2007). Captive breeding and reproductive biology of the *Protobothrops cornutus* and *Triceratolepidophis sieversorum*. *Russian Journal of Herpetology*, 14(1), 57–64.

De Silva, A. & Toriba, M. (1984). Reproductive habits of *Hypnale hypnale* (Merrem) in Sri Lanka. *The Snake*, 16, 135–138.

Slejipen, F. (1991). Breeding *Nerodia fasciata*. *Litteratura Serpentium*, 11(2), 39–43.

Smith, M.A. (1951). *The British Amphibians & Reptiles*. London: Collins.

Solórzano, A. & Cerdas, L. (1989). Reproductive biology and distribution of the terciopelo, *Bothrops asper* Garman (Serpentes: Viperidae), in Costa Rica. *Herpetologica*, 45(4), 444–450.

Street, D. (1979). *The Reptiles of Northern and Central Europe*. London: B. T. Batsford.

Termeer, M. (1991). Breeding Results: *Elaphe obsoleta quadrivittata* (F2). *Litteratura Serpentium*, 11(5), 115.

Tryon, B.W. (1979). Reproduction in captive forest cobras, *Naja melanoleuca* (Serpentes: Elapidae). *Journal of Herpetology*, 13(4), 499–504.

Tryon, B.W. & Carl, G. (1980). Reproduction in the mole kingsnake, *Lampropeltis calligaster rhombomaculata* (Serpentes, Colubridae). *Transactions of the Kansas Academy of Science (1903-)*, 83(2), 66–73.

Tryon, B.W. & Murphy, J.B. (1982). Miscellaneous notes on the reproductive biology of reptiles. 5. Thirteen varieties of the Genus Lampropeltis, Species *mexicana, triangulum* and *zonata*. *Transactions of the Kansas Academy of Science (1903-)*, 85(2), 96–119.

Tu, M.C., Lillywhite, H.B., Menon, J.G., & Menon, G.K*.* (2002). Postnatal ecdysis establishes the permeability barrier in snake skin: New insights into barrier lipid structures. *Journal of Experimental Biology*, 205(19), 3019–3030. https://doi.org/10.1242/jeb.205.19.3019.

Valencia, J.H. & Garzon-Tello, K. (2018). Reproductive behavior and development in *Spilotes sulphureus* (Serpentes: Colubridae) from Ecuador. *Phyllomedusa*, 17(1), 113–126. https://doi.org/10.11606/issn.2316-9079.v17i1p113-126.

Walsh, T. & Murphy, J.B. (2003). Observations on the husbandry, breeding and behaviour of the Indian python. *International Zoo Yearbook*, 38(1), 145–152. https://doi.org/10.1111/j.1748-1090.2003.tb02074.x.

Wastell, A.R. & Mackessy, S.P. (2016). Desert massasauga rattlesnakes (*Sistrurus catenatus edwardsii*) in Southeastern Colorado: Life history, reproduction, and communal hibernation. *Journal of Herpetology*, 50(4), 594–603. https://doi.org/10.1670/15-084.

Wharton, C.H. (1960). Birth and behavior of a brood of cottonmouths, *Agkistrodon piscivorus piscivorus* with notes on tail-luring. *Herpetologica*, 16(2), 125–129.

Wray, K.P., Margres, M.J., Seavy, M., & Rokyta, D.R*.* (2015). Early significant ontogenetic changes in snake venoms. *Toxicon*, 96, 74–81. https://doi.org/10.1016/j.toxicon.2015.01.010.
